# Different roles of integrin-β1 and integrin-αv for type IV secretion of CagA versus cell elongation phenotype and cell lifting by *Helicobacter pylori*

**DOI:** 10.1371/journal.ppat.1008564

**Published:** 2020-06-04

**Authors:** Wolfgang Fischer, Rainer Haas

**Affiliations:** 1 Medical Microbiology and Hospital Epidemiology, Max von Pettenkofer Institute, Faculty of Medicine, Ludwig-Maximilians-University Munich, Munich, Germany; 2 German Center for Infection Research (DZIF), Ludwig-Maximilians-University Munich, Munich, Germany; University of Illinois, UNITED STATES

The mechanism for delivery of the cytotoxin-associated gene A (CagA) effector protein into eukaryotic host cells by the *cag*-Type IV secretion system (*cag*-T4SS) of *Helicobacter pylori* is still not well understood. Two different ligand–receptor pairs have been described to be involved in this process: interaction of several Cag proteins, including CagL, with different integrin heterodimers, such as α5β1 and αVβ6 integrins [1–3], and interaction of the *H*. *pylori* outer membrane protein HopQ with carcinoembryonic antigen-related cell adhesion molecules (CEACAMs) [4, 5].

Analysing the relative importance of both receptor families using CRISPR/Cas9 gene knockout AGS and KatoIII cell lines, we found that neither β1 nor any other integrin heterodimers on the surface of gastric epithelial cells were necessary for CagA translocation, but distinct CEACAM receptors proved to be essential [6]. This finding was unexpected and in contrast to a number of earlier publications, which had assumed an essential mechanistic role of integrins for CagA translocation.

The formal comment by Tegtmeyer and Backert now reports on experiments performed with a different set of *H*. *pylori* strains than those used in our laboratory, but the same AGS integrin knockout cells. These experiments first verified the integrin knockout status of the AGS cells by immunoblot and then confirmed the results of the Zhao and colleagues paper concerning the independence of CagA translocation and phosphorylation from integrins. In the following, they extended their research with the wild-type and knockout cell lines by looking at cell elongation, also known as the hummingbird phenotype. This cell elongation can be (and has frequently been) used to visualise the effect of translocated and phosphorylated CagA in this particular cell line (notably, it is not observed in other cell lines of gastric or non-gastric origin besides AGS [7]). By quantification of the data, they show that the integrin β1 knockout (ITGB1) AGS cell line did not develop the hummingbird phenotype, whereas inactivation of αV and β4 integrins (ITGAvB4 knockout cells) enhanced this phenotype significantly upon infection with *H*. *pylori*.

In our study, we had not noted any obvious morphological differences between AGS wild-type and integrin knockout cells, but we had to grow ITGAvB4 knockout cells in collagen-containing media in order to keep them adherent. Given that the hummingbird phenotype results from a retraction defect caused by focal adhesions in these cells, we cannot exclude that changes in the presence of integrin (or also addition of collagen to the cultivation media) might cause an altered behavior with respect to focal adhesions, as also pointed out in the formal comment by Tegtmeyer and Backert. We did not initially quantify the hummingbird phenotype in our study. After thorough reinspection of our previous data, we still conclude that ITGB1 knockout AGS cells are able to form elongated cells, but to a lesser extent than wild-type cells. We also see a tendency towards stronger morphological changes for the ITGAvB4 knockout cells; however, we also note the presence of some irregularly shaped cells already without infection, which we find difficult to distinguish from genuine (CagA-induced) hummingbird cells. In conclusion, we believe that the data shown in the formal comment by Tegtmeyer and Backert are not contradictory to our original data, but just represent earlier or less pronounced stages of the cell reshaping process induced by intracellular phosphorylated CagA.

## References

[1] Kwok T, Zabler D, Urman S, Rohde M, Hartig R, Wessler S, et al *Helicobacter* exploits integrin for type IV secretion and kinase activation. Nature. 2007;449(7164):862–6. 10.1038/nature06187 17943123

[2] Jimenez-Soto LF, Kutter S, Sewald X, Ertl C, Weiss E, Kapp U, et al *Helicobacter pylori* type IV secretion apparatus exploits beta1 integrin in a novel RGD-independent manner. PLoS Pathog. 2009;5(12):e1000684 10.1371/journal.ppat.1000684 19997503 PMC2779590

[3] Buss M, Tegtmeyer N, Schnieder J, Dong X, Li J, Springer TA, et al Specific high affinity interaction of *Helicobacter pylori* CagL with integrin alphaV beta6 promotes type IV secretion of CagA into human cells. Febs J. 2019;286(20):3980–97. Epub 2019/06/15. 10.1111/febs.14962 31197920

[4] Königer V, Holsten L, Harrison U, Busch B, Loell E, Zhao Q, et al *Helicobacter pylori* exploits human CEACAMs via HopQ for adherence and translocation of CagA. Nat Microbiol. 2016;2:16188–99. nmicrobiol2016188 [pii]; 10.1038/nmicrobiol.2016.188 27748756

[5] Javaheri A, Kruse T, Moonens K, Mejias-Luque R, Debraekeleer A, Asche CI, et al *Helicobacter pylori* adhesin HopQ engages in a virulence-enhancing interaction with human CEACAMs. Nat Microbiol. 2016;2:16189 nmicrobiol2016189 [pii]; 10.1038/nmicrobiol.2016.189 27748768

[6] Zhao Q, Busch B, Jimenez-Soto LF, Ishikawa-Ankerhold H, Massberg S, Terradot L, et al Integrin but not CEACAM receptors are dispensable for *Helicobacter pylori* CagA translocation. PLoS Pathog. 2018;14(10):e1007359 Epub 2018/10/27. 10.1371/journal.ppat.1007359 30365569 PMC6231679

[7] Bauer B, Moese S, Bartfeld S, Meyer TF, Selbach M. Analysis of cell type-specific responses mediated by the type IV secretion system of *Helicobacter pylori*. Infect Immun. 2005;73(8):4643–52. 10.1128/IAI.73.8.4643-4652.2005 16040977 PMC1201271

